# Dissecting and stimulating instruments in thyroid surgery: principles, applications and clinical role

**DOI:** 10.3389/fmedt.2026.1845958

**Published:** 2026-08-17

**Authors:** Francesco Brucchi, Carla Colombo, Giuseppe Mercante, Paolo Battaglia, Pietro Indelicato, Diego Barbieri, Davide Lombardi, Gabriele Materazzi, Gianlorenzo Dionigi

**Affiliations:** 1 Division of General Surgery, Istituto di Ricovero e Cura a Carattere Scientifico (IRCCS) Istituto Auxologico Italiano, Milan, Italy; 2 Department of Pathophysiology and Transplantation, University of Milan, Milan, Italy; 3 Division of Endocrinology, Istituto di Ricovero e Cura a Carattere Scientifico (IRCCS) Istituto Auxologico Italiano, Milan, Italy; 4 Department of Otolaryngology-Head and Neck Surgery, IRCCS Azienda Ospedaliero-Universitaria Di Bologna, Bologna, Italy; 5 Dipartimento di Scienze Mediche e Chirurgiche, Alma Mater Studiorum-Università di Bologna, Bologna, Italy; 6 Division of Otorhinolaryngology, Department of Biotechnology and Life Sciences, University of Insubria, ASST Lariana, Como, Italy; 7 Division of Head and Neck, San Raffaele Scientific Institute IRCCS (Istituto Di Ricovero E Cura a Carattere Scientifico), Otorhinolaryngology Unit, Milan, Italy; 8 School of Medicine, Vita-Salute San Raffaele University, Milan, Italy; 9 Unit of Otorhinolaryngology-Head and Neck Surgery, ASST Spedali Civili di Brescia, Department of Medical and Surgical Specialties, Radiologic Sciences and Public Health, University of Brescia, Brescia, Italy; 10 Department of Surgical, Medical, Molecular Pathology and Critical Area, Unit of Endocrine Surgery, Pisa University Hospital, Pisa, Italy

**Keywords:** intraoperative neuromonitoring, IONM, parathyroid surgery, recurrent laryngeal nerve, SDI, stimulating dissecting instrument, thyroid surgery

## Abstract

Stimulating dissecting instruments (SDIs) combine fine dissection and calibrated nerve stimulation in a single tool, addressing the workflow limitations of conventional intermittent intraoperative neuromonitoring (IONM) in thyroid and parathyroid surgery. By eliminating repeated exchanges between a dissecting instrument and a separate stimulator, SDIs enable quasi-continuous functional assessment of the recurrent laryngeal nerve (RLN) and external branch of the superior laryngeal nerve (EBSLN) precisely during high-risk maneuvers, such as Berry's ligament dissection and superior pole management. Prospective clinical data in open thyroidectomy show that SDIs are feasible, safe, and effective in detecting early traction-related EMG changes, allowing timely release of stress and resulting in very low rates of postoperative RLN palsy, while experimental endoscopic and transoral models confirm reliable dose–response characteristics at clinically acceptable stimulation thresholds. In minimally invasive and narrow-corridor approaches, SDIs reduce instrument traffic and maintain neuromonitoring without disrupting operative flow, and their real-time feedback enhances training by directly linking dissection quality to neurophysiological signals. Within current guideline-based IONM frameworks, SDIs represent a practical evolution toward integrated, high-frequency monitoring, with future development expected to focus on their combination with energy-based devices, advanced imaging, and intelligent EMG analysis.

## Introduction

Thyroid surgery is among the most frequently performed endocrine operations worldwide (1). Protecting the recurrent laryngeal nerve (RLN) and the external branch of the superior laryngeal nerve (EBSLN) remains a central challenge, as reported RLN injury rates range from well below 3% in optimized series to over 12% in high-risk or reoperative settings (2).

Over the past two decades, intraoperative neuromonitoring (IONM) has evolved from an experimental adjunct to a widely adopted safety measure in thyroidectomy (3). National datasets show its use in the majority of cases and demonstrate a statistically significant reduction in RLN injury odds after adjustment for confounders (4).

Despite this widespread use, conventional intermittent IONM has an inherent workflow limitation: the surgeon must repeatedly interrupt dissection to exchange the active instrument for a dedicated handheld stimulator, trigger the stimulus, interpret the electromyographic (EMG) response, and then return to the dissecting tool (5). This cycle is particularly frequent during dissection around the Berry ligament and RLN skeletonization, where each instrument change results in both a time penalty and a monitoring gap during which evolving traction or thermal injury may occur without immediate EMG feedback (6).

To address this limitation, two broad families of techniques have been developed: continuous vagal nerve monitoring systems, which deliver automatic periodic stimulation via an electrode encircling the vagus nerve (C-IONM), and “quasi-continuous” solutions based on stimulating dissection instruments (SDI) that integrate dissection and stimulation, along with ancillary tools such as anchor stimulating devices and nerve trend analysis software (Table 1) (7–12). Continuous vagal monitoring requires placement of a dedicated electrode on the vagus nerve proximal to RLN branching, followed by regular stimulation – typically 10–60 times per minute with currents around 1–2 mA – resulting in sustained EMG acquisition from laryngeal muscles throughout the operation (13).

**Table 1:** Strengths and limitations of intermittent, quasi-continuous, and continuous IONM during thyroidectomy.

Modality	Concept	Strengths	Limitations
Intermittent IONM (handheld stimulator)	Surgeon-initiated, on-demand stimulation of RLN or vagus with a separate probe	Widely available; simple workflow; low incremental cost; allows mapping and functional checks at predefined steps	Requires repeated instrument exchange and interruption of dissection; monitoring gaps between stimulations; evolving traction or thermal injury may occur without immediate EMG feedback; timing and frequency operator-dependent
Quasi-continuous IONM: stimulating dissection instruments (SDI)	Stimulation integrated into the dissecting tool (e.g., suction, spatula, dissector)	Reduces need to switch between dissecting and stimulating instruments; facilitates frequent “on-the-fly” testing near Berry ligament and during RLN skeletonization; may improve efficiency and surgeon comfort	Stimulation only when the active instrument contacts tissue; monitoring not truly continuous; performance depends on how consistently the instrument is used; device availability and costs vary; learning curve for safe use of stimulating tips

Multiple clinical series and experimental studies indicate that C-IONM reliably detects impending RLN compromise by identifying characteristic EMG changes – most commonly progressive amplitude decline and latency prolongation – often related to traction on the thyroid lobe or nerve, thus allowing immediate modification of the surgical maneuver before permanent damage occurs (14–16). Concerns regarding potential vagal nerve injury from continuous stimulation have been addressed by recent prospective studies showing that stimulation at recommended settings does not produce clinically relevant deterioration in proximal or distal EMG amplitudes along the vagus-RLN axis (13). Nevertheless, C-IONM adds procedural complexity, requires vagal nerve exposure and electrode placement, and entails specific device costs, so its use is most often advocated in high-risk thyroidectomies such as reoperations, invasive malignancies, or bilateral procedures where staged surgery is considered (13).

In parallel, quasi-continuous monitoring strategies have been developed to maintain near-real-time feedback without permanent vagal electrodes, primarily through the introduction of SDIs that combine the functions of fine dissection and calibrated nerve stimulation at the tip of the same instrument (10–12). With SDIs, every contact between the forceps and perithyroidal tissue can be transformed into a test of RLN integrity at the surgeon's discretion, substantially reducing or eliminating the need for separate probe exchanges and minimizing unmonitored intervals during critical steps (10–12).

The seminal prospective study by Chiang and colleagues enrolled 100 consecutive patients (168 nerves at risk) to evaluate early prototypes of SDIs used during the high-risk phases of RLN dissection (10). In that series, SDI application was feasible in all cases and did not introduce any device-related morbidity; substantial traction-related EMG deterioration (defined as more than 50% amplitude reduction) was detected in 19 nerves, and prompt release of thyroid traction led to gradual EMG recovery, with only one nerve ultimately developing temporary postoperative vocal cord palsy despite a 79% amplitude drop (10). These findings demonstrated that SDIs can provide immediate, context-specific EMG feedback precisely at the moment and site of mechanical stress, functioning as an early warning system analogous to C-IONM but without the need for vagal electrode placement (10).

Subsequent experimental work has expanded the SDI concept to endoscopic and transoral approaches, confirming that endoscopic stimulating dissectors can safely monitor both vagus and RLN function during transoral endoscopic thyroidectomy, although higher current intensities may be required compared with open handheld probes, and tip-end stimulation is generally more efficient than lateral-tip modes (11). In addition to these hardware innovations, software tools such as advanced nerve trend displays, alarm algorithms, and anchor stimulation protocols (which standardize periodic stimulation at fixed reference points) have further enhanced quasi-continuous monitoring by enabling visualization and quantification of subtle EMG trends over time, rather than relying solely on isolated spot checks (9–12).

In this context, the SDI concept – typically a fine forceps capable of both sharp and blunt dissection and graded stimulation – has emerged as a practical solution to the workflow bottleneck of intermittent IONM, particularly in open thyroid and parathyroid surgery, where instrument ergonomics and tactile feedback are critical (10–12). By integrating stimulation into the primary dissecting tool, SDIs address many of the monitoring gaps inherent in conventional intermittent systems, while avoiding some of the invasiveness and setup requirements of vagal C-IONM (10–12). Their effectiveness in early traction-injury detection has been demonstrated in both clinical and experimental settings (10–12).

Within this evolving paradigm, the present paper focuses on SDI technology, summarizing its engineering foundations, electrophysiological characteristics, operative technique, clinical performance, and expanding applications across open, endoscopic, and transoral thyroid and parathyroid procedures. In doing so, it positions SDIs and related quasi-continuous monitoring tools within the broader spectrum of modern RLN and EBSLN protection strategies, from traditional intermittent IONM to fully continuous vagal systems, with the goal of optimizing nerve safety while maintaining operative efficiency.

## Beyond thyroid surgery

Stimulating dissection instruments (SDIs) can extend real-time nerve monitoring far beyond thyroid and parathyroid surgery. By combining precise dissection and calibrated stimulation in a single tool, they enable functional nerve mapping without repeated instrument changes or interruptions to the operative flow.

In general surgery, SDIs may support identification and preservation of pelvic autonomic nerves during total mesorectal excision, critical plexus branches in complex hepatobiliary or pancreatic dissections, and lumbar plexus or sympathetic fibers during adrenal and retroperitoneal surgery. In minimally invasive laparoscopy and robotics, long, insulated SDIs with articulated tips could provide near-continuous neuromonitoring through the working instrument, overcoming a major limitation of handheld intermittent probes (17–20).

The same principle applies to other high-risk fields: in neck dissection, SDIs may facilitate monitoring of facial, spinal accessory, and marginal mandibular branches; in parotid surgery, they could aid preservation of facial nerve branches in both primary and revision cases; and in thoracic procedures, they may assist in assessing vagus and phrenic nerve function during mediastinal or hilar dissection. In all these scenarios, SDIs replace the traditional dissection–stimulation “handoff” with a continuous, integrated workflow, allowing every critical maneuver to be performed with an instrument that provides immediate functional feedback on nerves at risk (17–20).

## SDI: design, engineering, and electrophysiological principles

### Concept and historical development

The concept of stimulating dissection instruments (SDIs) arose from the observation that the highest-risk moments in IONM-assisted thyroidectomy occur when the surgeon is actively dissecting and a handheld stimulating probe is not in use, resulting in intervals without electromyographic (EMG) feedback (Table 2) (7). The first SDI prototypes, described by Chiang and colleagues in 2015, adapted standard dissecting forceps to interface with the IONM stimulator cable, effectively transforming a routine instrument into a real-time electrophysiological sensor without fundamentally altering the dissection technique (10).

**Table 2:** Surgical applications of stimulating dissection instruments (SDIs) in thyroid and parathyroid surgery.

Procedure	Target nerves	SDI-assisted maneuvers	Potential benefits
Hemithyroidectomy/lobectomy	RLN, EBSLN	Integrated dissection and stimulation along the tracheoesophageal groove and superior pole; mapping of RLN entry point and EBSLN during vessel ligation	Earlier identification of RLN/EBSLN; reduced traction or thermal injury; safer superior pole control
Total thyroidectomy	RLN, EBSLN, VN (via vagal stimulation)	Systematic “on-the-fly” mapping during bilateral RLN skeletonization; superior pole dissection with EBSLN testing; intermittent vagal stimulation through SDI or separate electrode	Continuous functional feedback during high-risk steps; facilitation of staged thyroidectomy in case of signal deterioration
Thyroidectomy with central neck dissection	RLN, VN	SDI-guided dissection in paratracheal and tracheoesophageal spaces; mapping of RLN branches and nerves at risk within nodal tissue	Safer clearance around the RLN; reduced inadvertent nerve branch injury during nodal dissection
Reoperative thyroid or parathyroid surgery	RLN, VN	Dissection through scarred tissue planes with simultaneous stimulation; confirmation of RLN course before and after fibrotic adhesiolysis	Improved localization of distorted RLN; lower risk in dense fibrosis or post-ablative fields
Parathyroidectomy (focused or bilateral)	RLN, VN	Mapping of RLN during exploration; confirmation of nerve integrity after parathyroid excision or autotransplantation	Decreased risk of RLN injury in targeted or reoperative parathyroid surgery
Neck dissection associated with thyroid/parathyroid procedures	Spinal accessory nerve, marginal mandibular, cervical branches (as applicable)	Use of SDI during lymphadenectomy to test motor branches encountered in the field	Additional protection of non-laryngeal cervical motor nerves in combined oncologic procedures

Building on this proof of concept, commercial SDI systems (e.g., Beckdal, Songjiang, Shanghai, China) have developed into CE-marked devices with standardized technical features. These instruments are produced in familiar hemostatic configurations (such as curved-tip and Mixter/right-angle), incorporate PTFE insulation along the shaft, and use a medical-grade conductive tip to focus the stimulus at the site of tissue contact. Integration with neuromonitoring units is achieved via a standard DIN 42802 connector, ensuring compatibility with major IONM platforms and allowing incorporation into existing operative workflows with minimal disruption.

### Instrument architecture

**Figure 1:**
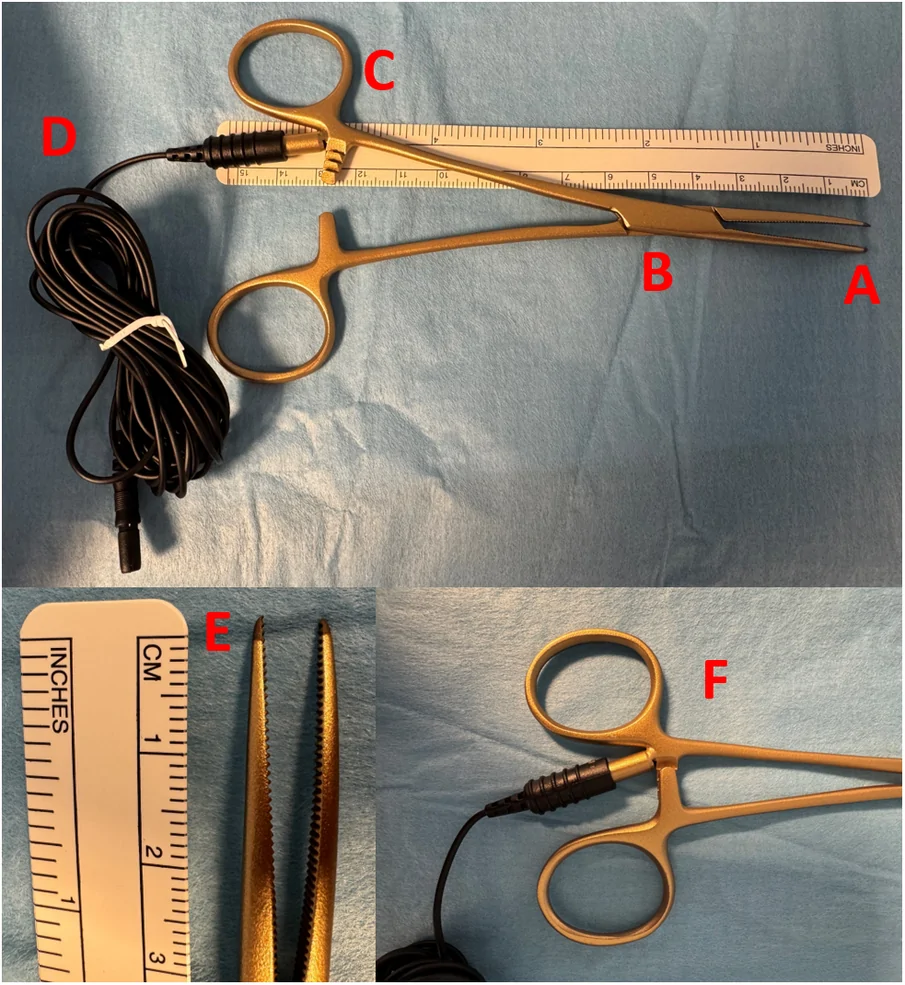
Stimulating dissection instrument (SDI-TEC): design and key functional features. The SDI is a surgeon-driven device that combines an EMG stimulating probe with dissecting forceps for intraoperative neuromonitoring of the recurrent laryngeal nerve during thyroid surgery. **(A)** Fine, tapered, serrated jaws enable delicate grasping, blunt dissection, and direct nerve stimulation at the tip. **(B)** Long, slim, gently curved shaft provides extended reach and precise access to deep or narrow operative fields. **(C)** Standard ring-handle and ratchet lock mechanism allow controlled closure pressure and stable instrument positioning. **(D)** Integrated insulated stimulation cable is connected to the instrument, using a universal connector with standard DIN 42802 plugs to ensure compatibility with most neuromonitoring systems. **(E)** Close-up view of the atraumatic, serrated tip profile demonstrates the narrow working surface designed to optimize tissue handling while concentrating the stimulation current at a single monopolar active tip; available in curved or right-angle configurations to facilitate precise dissection and real-time nerve mapping within the thyroid bed. **(F)** Proximal handle region and insulated shaft show the ergonomic, surgeon-driven hemostat-style handle and the surgical-grade metal shaft with PTFE (Polytetrafluoroethylene) insulation that maintains electrical isolation along the instrument, while a reinforced junction between the cable and metal body provides strain relief and electrical safety.

The SDI is organized into four main functional zones (Figure 1). The proximal handle provides an ergonomic grip compatible with standard operative hand positioning and incorporates the electrical interface that connects the instrument to the neuromonitoring system. The intermediate shaft is coated with high-grade PTFE insulation, ensuring complete electrical isolation along the instrument body so that stimulation current is confined to the distal extremity and does not disperse to adjacent tissues.

The active distal tip consists of uninsulated conductive metal at the jaws, which delivers the electrical stimulus directly to tissue in contact with or adjacent to the nerve. Two stimulation configurations are available: tip-end mode, in which current is emitted from the terminal extremity of the jaws, and tip-lateral mode, in which current is delivered from the lateral surface of the instrument. Electrophysiological testing indicates that tip-end stimulation achieves maximal EMG responses at slightly lower current intensities than lateral-tip stimulation. This integrated architecture allows the surgeon to apply gentle traction, perform blunt dissection of fascial planes, while simultaneously sampling neural structures at each contact and converting routine maneuvers into opportunities for real-time functional nerve assessment.

### Dimensions of SDI

The Beckdal stimulating dissection instruments used in this study are monopolar stimulation dissection forceps available in both fine curved-tip and Mixter (right-angle) configurations, each offered in five overall lengths (125, 140, 160, 180, and 200 mm). The range of sizes allows the surgeon to select a shorter instrument for standard open thyroidectomy and longer shafts for deep or retrosternal dissection, while maintaining familiar ergonomics comparable to conventional hemostatic forceps (Table 3). All models share the same functional architecture, with a fully insulated shaft and a small exposed distal segment that delivers the electrical stimulus precisely at the tip–tissue interface. The instruments connect to the neuromonitoring system via a standard DIN-compatible cable, enabling their use as stimulating dissectors without altering the existing IONM platform or the surgeon's basic dissection technique.

**Table 3:** Stimulating dissection forceps configurations.

Item code	Configuration	Overall length (mm)
SI000013	Fine curved tip	125
SI000014	Fine curved tip	140
SI000016	Fine curved tip	160
SI000018	Fine curved tip	180
SI000020	Fine curved tip	200
SI100013	Mixter (right-angle)	125
SI100014	Mixter (right-angle)	140
SI100016	Mixter (right-angle)	160
SI100018	Mixter (right-angle)	180
SI100020	Mixter (right-angle)	200

### Electrophysiological performance

Quantitative EMG performance of SDI technology was systematically evaluated by Zhang et al. (11). The study demonstrated a consistent dose–response relationship, with EMG amplitude increasing progressively as stimulation current increased across all stimulation configurations. VN stimulation thresholds were 0.28 mA for the conventional hand probe, 0.56 mA for SDI tip-end, and 0.58 mA for SDI tip-lateral stimulation, with no statistically significant difference between the two SDI modes (*P* = 0.11). RLN maximal response thresholds were correspondingly higher for SDI compared with the hand probe—0.60 mA (hand probe) versus 0.95 mA (tip-end) and 1.05 mA (tip-lateral)—again without a significant difference between SDI configurations (*P* = 0.31). Latency values remained statistically equivalent among all groups, and prolonged supramaximal stimulation (>5 mA for more than 10 min) did not induce measurable functional or structural nerve damage, supporting the safety of repetitive SDI activation (11). Collectively, these data indicate that SDIs function within clinically acceptable electrophysiological ranges; the modestly higher thresholds relative to direct hand probes are attributable to increased impedance from the metallic instrument–tissue interface and interposed fascial layers. In routine clinical use, SDIs are therefore operated at 1–3 mA, aligning with standard IONM settings while compensating for this threshold shift and ensuring reliable nerve activation. Thus, the EMG data are comparable to those obtained with other commonly used stimulation devices (Table 4).

**Table 4:** Comparative technical and electrophysiological profile of conventional probe versus SDI configurations.

Parameter	Conventional Dissector + Hand Probe	SDI (Open Surgery)	Endoscopic SDI (MIS/TOETVA)
Instrument changes intraop.	Frequent (every stimulation)	None	None
Real-time EMG feedback	Delayed/intermittent	Continuous during dissection	Continuous during dissection
Stimulation threshold (VN)	∼0.28 mA (hand probe)	∼0.56 mA (tip-end)	∼0.58 mA (tip-lateral)
RLN max response threshold	∼0.60 mA	∼0.95 mA (tip-end)	∼1.05 mA (tip-lateral)
Morbidity from device	None documented	None documented	None documented
Learning curve	Moderate (probe handling)	Short	Moderate (endoscopic skills)
Neuromonitoring system compatibility	System-specific	Universal (DIN 42802 plug)	Universal (DIN 42802 plug)

VN, vagus nerve; RLN, recurrent laryngeal nerve; DIN, Deutsche Industrie-Norm; TOETVA, transoral endoscopic thyroidectomy vestibular approach.

In the dose–response experiments by Zhang et al., stimulation thresholds of 0.95 mA for the SDI tip-end and 1.05 mA for the SDI tip-lateral were defined as the lowest currents at which a stable plateau of RLN-evoked EMG amplitude was achieved, i.e., further current increments did not produce a clinically relevant amplitude gain (11). At these threshold currents, the corresponding mean RLN-evoked EMG amplitudes were approximately 1,182 ± 197 *μ*V (tip-end) and 1,155 ± 189 μV (tip-lateral), as recorded at the 1.0 mA stimulation level (Table 4). These values lie well above the INMSG-recommended baseline threshold of >500 μV and are fully comparable to those obtained with conventional hand-held stimulating probes under standard IONM settings. Latency values remained statistically unchanged across the entire current spectrum and were not affected by prolonged supramaximal stimulation, confirming that intraoperative risk stratification relies primarily on amplitude behavior (absolute values and percentage change from baseline), while latency serves as a supportive but less discriminative parameter. Accordingly, SDI-guided monitoring applies the same interpretive amplitude criteria recommended by the International Neural Monitoring Study Group (e.g., absolute amplitude ≥100 μV and ≥50% amplitude decline as thresholds for clinically relevant deterioration) to the high-quality EMG signals routinely observed at 1–3 mA stimulation.

### Insulation and safety

Electrical safety is a fundamental engineering principle in the design of SDI technology. The instrument shaft is coated with PTFE insulation, which provides high dielectric strength and structural stability during continuous exposure to conductive media such as saline and blood, thereby maintaining consistent electrical isolation along the device throughout the procedure (21, 22). The stimulation current is confined to the short, uninsulated distal tip, enabling a spatially precise activation zone that minimizes current diffusion within the operative field and reduces the risk of unintended stimulation of adjacent neural structures, including the RLN and EBSLN, during dissection and mobilization. This spatially restricted current emission is especially advantageous in confined, fluid-filled surgical corridors, where uncontrolled current propagation could otherwise pose a hazard. Consistent with this design rationale, clinical experience with SDI-assisted IONM has shown no evidence of device-related neural injury, supporting the conclusion that SDIs provide accurate functional monitoring while maintaining procedure-specific neurophysiological safety (10–12). Because the two arms of the SDI are joined by a central hinge, they are electrically continuous and function as a single active (stimulating) electrode, with the stimulation circuit completed through a separate dispersive return (ground) electrode placed on the patient, exactly as in a conventional monopolar stimulating probe. Effective current delivery therefore depends on the quality of contact between the monopolar active tip and the target tissue rather than on bridging of tissue between separate poles. Because a single point of good tip–tissue contact is sufficient to deliver the full programmed current, wide opening of the jaws during dissection does not by itself compromise stimulation; adequate responses are ensured by contact quality and current level rather than by jaw closure. To preserve reliable current density, stimulation currents should be set toward the upper end of the clinically acceptable range (2–3 mA) in high-impedance situations such as scarred planes or fluid-filled corridors. Under these conditions, experimental and clinical data indicate that SDIs maintain reliable activation of vagus and RLN signals without evidence of device-related neural injury, even when supramaximal currents are applied for prolonged periods.

## SDI in the context of surgical neuromonitoring

### Guidelines and standardization

The practice of intraoperative neuromonitoring (IONM) in thyroid and parathyroid surgery is guided by the consensus recommendations of the International Neural Monitoring Study Group (INMSG) (23, 24). These guidelines establish standardized nomenclature (e.g., signal events, loss-of-signal thresholds), minimum performance requirements for monitoring systems, and define the procedural sequence of IONM testing (V1–R1–R2–V2), along with algorithmic responses to loss-of-signal (LOS) events (23, 24). The principal electrophysiological endpoints include: (1) EMG amplitude, with signals ≥100 µV classified as “present” and a decline >50% from baseline considered a clinically significant deterioration; (2) latency, where prolongation combined with amplitude reduction suggests early neuropraxia; and (3) LOS, defined by an EMG amplitude <100 µV and interpreted as presumptive functional nerve injury, prompting immediate intraoperative troubleshooting and, in bilateral cases, consideration of a staged procedure. Within this regulatory and procedural framework, SDIs represent a technological advancement that implements and reinforces these guideline principles by integrating standardized stimulation capability into the primary dissecting instrument (Table 5) (23, 24). This configuration increases both the frequency and spatial resolution of EMG signal acquisition without interrupting the surgical flow. By transforming each dissection step into a potential checkpoint for guideline-concordant EMG validation – using the same INMSG-defined criteria for signal adequacy, deterioration, and LOS – SDIs enable continuous, protocol-driven nerve function assessment. This functional integration may improve intraoperative decision-making regarding traction modulation, side-to-side functional comparison, and surgical staging strategy. Contemporary registry and cohort analyses show that routine IONM adoption continues to expand globally and is associated with a modest but statistically significant reduction in recurrent laryngeal nerve (RLN) injury incidence overall, with relatively greater benefit observed in high-risk subgroups such as patients with locally advanced or malignant thyroid disease (3). Collectively, these findings underscore the dual importance of technological refinement and strict procedural standardization in optimizing the safety and reproducibility of nerve monitoring across diverse surgical settings.

**Table 5:** SDI integration into the standardized four-step IONM protocol: timing advantages and practical implications for nerve identification and protection.

IONM step/signal	Standard action and interpretation	SDI-specific advantage over conventional probe	Practical implication for nerve protection
V1 (vagus, pre-dissection)	Confirm system function; stimulate ipsilateral vagus at 1–2 mA; EMG ≥100 µV verifies tube position, electrode contact, and neuromuscular transmission.	SDI can elicit V1 during initial upper pole and carotid sheath mobilization, enabling earlier functional confirmation of vagal integrity directly within the thyroid bed.	Earlier V1 allows prompt correction of setup issues and EMG reliability before RLN manipulation; reduces delay before safe nerve handling.
R1 (RLN, pre-exposure)	Locate the RLN before full visual identification using the “cross-method”; stimulate perpendicular then parallel to the trachea to define course and detect non-recurrent variants.	Because the SDI acts as both dissector and stimulator, the RLN can be mapped and functionally identified earlier during initial tissue mobilization, shortening “blind” dissection time.	Earlier R1 reduces unmonitored dissection and may lower traction- or thermal-related RLN injury risk; aids intraoperative recognition of NRILN.
R2 (RLN, during dissection)	Apply stimulation during active tissue dissection at 1–2 mA; real-time EMG feedback guides maneuver; pause and release traction if amplitude decreases >50%; if LOS occurs, localize site and adjust strategy.	Continuous SDI use during perineural dissection provides high-frequency, quasi-continuous EMG interrogation at each tissue contact, enabling immediate detection of evolving amplitude decline or latency prolongation.	Transforms previously unmonitored intervals into actionable windows; promotes strict adherence to INMSG thresholds; increased granularity improves detection of evolving neuropraxia.
S1 (EBSLN, superior pole)	Stimulate EBSLN during superior pole exposure; assess CTM twitch and/or EMG response.	SDI detects S1 earlier while dissecting and mapping the cricothyroid–superior pole region, without dedicated probe exchange.	Earlier S1 shortens the interval in which the EBSLN is at risk during superior pole vessel ligation and control.
V2 (vagus, post-resection)	Final functional assessment of vagus/RLN before wound closure; amplitude <100 µV or LOS indicates significant injury; may justify staged procedure in bilateral disease.	SDI-guided mapping of RLN and EBSLN throughout the case increases confidence that any V2 deterioration reflects true neural injury rather than technical factors.	High prognostic value; improved reliability of final interpretation; V2 loss triggers laryngoscopy and supports staged surgery decisions in bilateral cases.

V, vagus nerve signal; R, RLN signal; S, EBSLN signal; LOS, loss of signal (<100 µV); CTM, cricothyroid muscle; NRILN, non-recurrent inferior laryngeal nerve; INMSG, International Neural Monitoring Study Group. SDI used primarily during Step 3 (R2) for real-time dissection monitoring, but provides timing advantages at all protocol steps.

### Intermittent vs. continuous IONM: where SDI fits

Two principal IONM paradigms are currently used in thyroid and parathyroid surgery. Intermittent IONM (I-IONM) relies on operator-initiated probe stimulation at predefined checkpoints (V1–R1–R2–V2). It is technically straightforward, relatively low cost, and widely accessible, but inherently leaves unmonitored intervals between stimulation events (5, 7). Continuous IONM (C-IONM) uses an electrode encircling the vagus nerve to deliver repetitive impulses throughout the procedure, enabling early detection of progressive EMG amplitude reduction and latency prolongation along the entire course of the RLN. However, this approach requires additional dissection within the carotid sheath, with potential manipulation of vascular and neural structures, longer setup time, and increased resource utilization (6).

**Figure 2:**
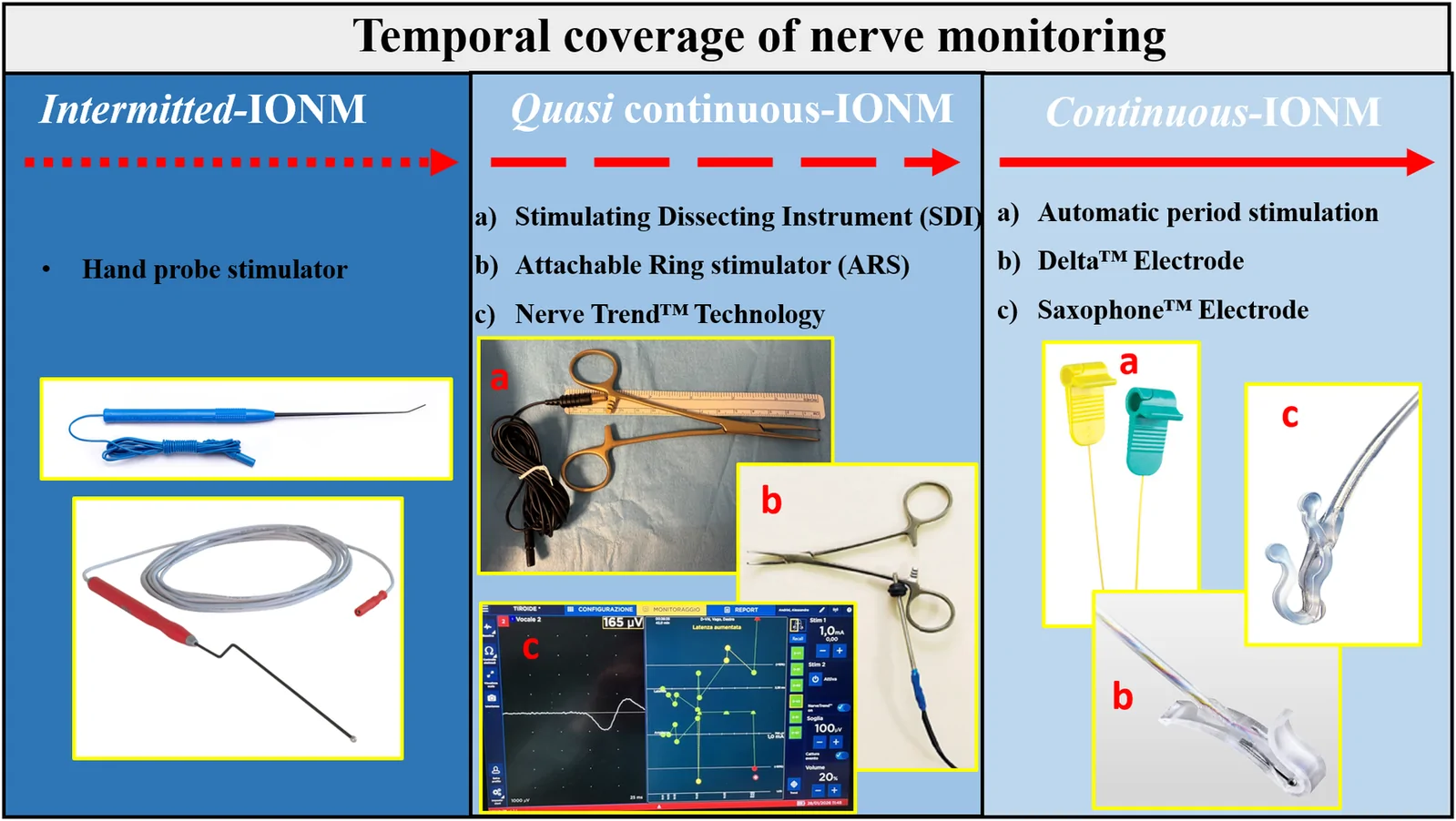
Temporal coverage of nerve monitoring with intermittent IONM, SDI-assisted quasi-continuous IONM, and continuous IONM.

Within this spectrum, SDI technology occupies an intermediate, “bridging” position between purely intermittent and fully continuous monitoring (Figure 2). By integrating stimulation capability into the primary dissecting instrument, SDIs convert each contact within the thyroid bed into an opportunity for real-time EMG interrogation, effectively transforming conventional I-IONM into a quasi-continuous modality focused on the thyroid lodge rather than on extrathyroidal vagal structures. This anatomically targeted monitoring offers two main advantages: it eliminates the need for carotid sheath dissection required for C-IONM electrode placement, thereby avoiding the theoretical risk of vascular or sympathetic chain injury, and it concentrates functional assessment precisely in the zones where mechanical and thermal insults to the RLN and EBSLN are most likely to occur, such as during Berry ligament division and lateral nerve skeletonization (10–12). In addition, SDI-based stimulation can be performed interchangeably by the primary surgeon or the assistant without altering the operative choreography, maintaining near-continuous feedback with minimal disruption of workflow. In settings where C-IONM is unavailable, impractical, or not routinely indicated, SDIs represent a cost-effective, anatomically focused enhancement of standard I-IONM, enabling high-frequency, guideline-concordant monitoring while preserving the simplicity of a conventional setup (Table 1).

## SDI in thyroid surgery: operative technique

### Pre-operative setup and anesthesia considerations

Preoperative setup and anesthetic management are critical determinants of the reliability of SDI-assisted IONM. An appropriately sized EMG endotracheal tube should be positioned with the surface electrodes precisely aligned against the true vocal folds, and correct placement must be verified by direct laryngoscopy and, when available, video laryngoscopy or fluoroscopy to ensure stable electrode–mucosa contact throughout the procedure (25). Neuromuscular blockade is limited to induction with an intermediate- or short-acting agent; no additional relaxant is administered after intubation, and any residual blockade, indicated by absent or markedly attenuated baseline EMG activity, is pharmacologically reversed – preferably with sugammadex – before monitoring is initiated. For anesthetic maintenance, total intravenous anesthesia (TIVA) with propofol and remifentanil is generally preferred, particularly in cases requiring multimodal neurophysiological monitoring such as additional motor evoked potentials, whereas low-concentration volatile anesthetics may be acceptable when only EMG-based IONM is used, provided they do not significantly depress EMG responsiveness (25). Before skin incision, electrode impedance is systematically measured and recorded, targeting values below 5 k*Ω* with an interelectrode difference of less than 1 k*Ω*, followed by acquisition of a stable baseline EMG signal (typically about 10 µV in the absence of stimulation), which confirms system integrity and provides a robust reference for interpreting subsequent changes in amplitude.

### Four-step IONM protocol with SDI integration

SDI can be seamlessly integrated into the standardized four-step IONM protocol (V1–R1–R2–V2) at Step 3 (R2 signal), which corresponds to the active dissection phase, without changing its structure or diagnostic criteria (23, 26). Instead of altering the guideline algorithm, SDIs reinforce it by embedding stimulation capability directly into the dissecting instrument, increasing the frequency and temporal precision of nerve assessment precisely when the RLN is most vulnerable (Table 5). Because the SDI also functions as a conventional dissector, V1 and R1 signals can be elicited earlier in the operation: the nerve may be identified anatomically during initial tissue mobilization and immediately confirmed functionally with the same instrument, eliminating the need for a separate stimulating probe (10, 23, 26). During the subsequent R2 phase, each relevant contact with perineural tissue can be associated with standardized stimulation and EMG recording, enhancing adherence to INMSG criteria for amplitude, latency, and loss of signal while preserving the familiar four-step framework. Thus, SDI use does not compromise standardization; it operationalizes and amplifies it, transforming the protocol from a series of isolated checkpoints into a high-density sequence of guideline-concordant assessments embedded throughout the dissection (23, 26).

## Operative technique: step-by-step SDI application

### Instrument connection and verification

**Figure 3:**
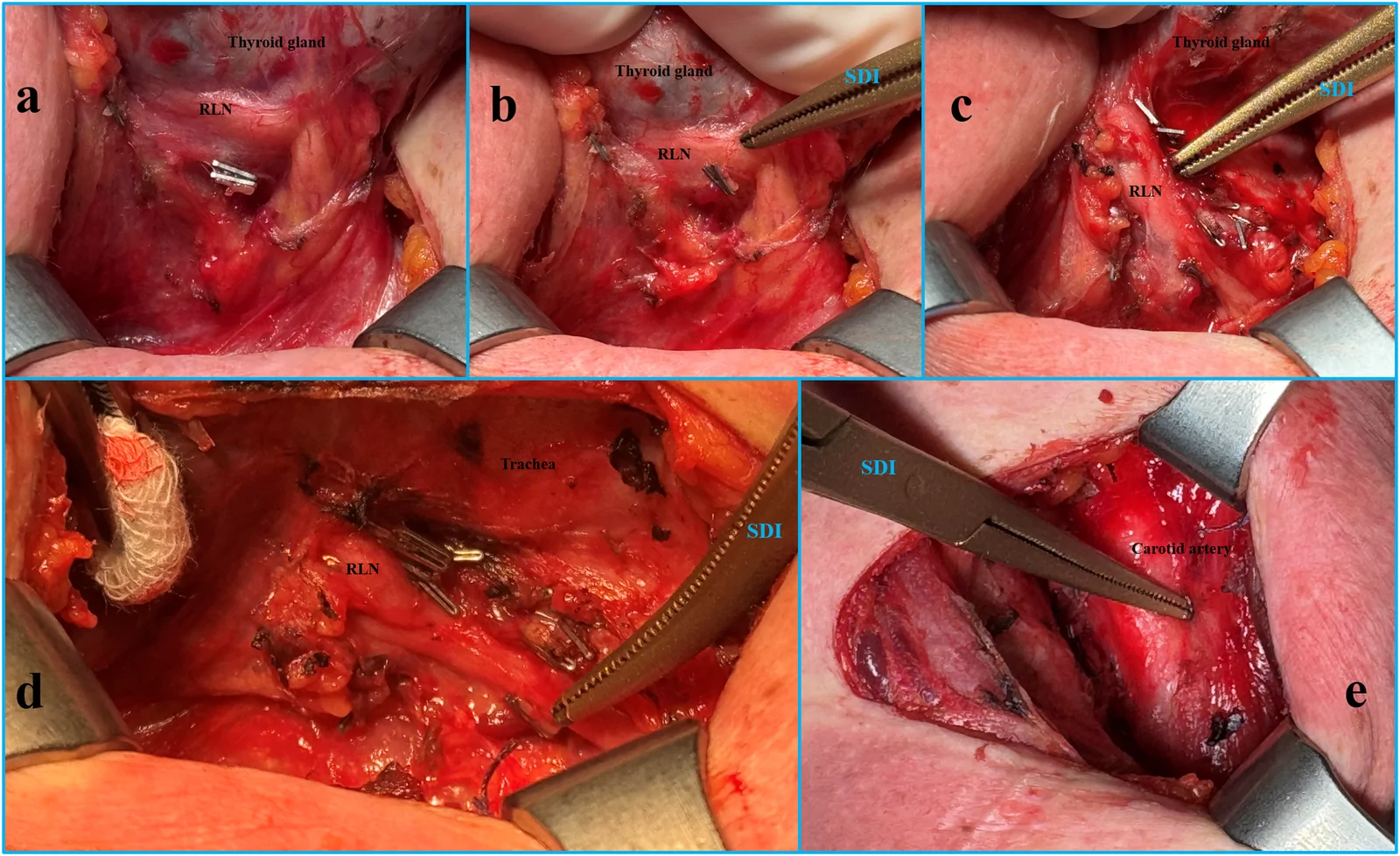
Usefulness of the SDI for meticulous RLN dissection and vagus nerve testing during TEC. **(a–d)** Stepwise use of the SDI enables careful, layer-by-layer dissection and simultaneous “quasi-continuous” stimulation of a recurrent laryngeal nerve (RLN) densely adherent to the thyroid gland, until complete mobilization and safe exposure are achieved **(d) (e)** Final vagus nerve stimulation within the opened carotid sheath: gentle SDI pressure on the carotid artery with 2–3 mA confirmed that the post-dissection vagal response (V2) was identical to the initial value (V1).

The SDI is connected to the stimulator output of the IONM system via a standard DIN 42802 connector before the skin incision. Correct integration of the instrument into the circuit is verified by eliciting an initial V1 response from the ipsilateral vagus nerve using the SDI at a current of 1–2 mA, prior to any thyroid manipulation (Figure 3). An evoked EMG amplitude of at least 100 µV confirms overall system integrity, including proper endotracheal tube positioning, satisfactory electrode–vocal fold contact, and effective neuromuscular transmission.

### Phase 1: lateral approach and initial dissection

During division of the middle thyroid vein and lateral mobilization of the thyroid lobe, the SDI is used for blunt dissection of fascial planes and isolation of vascular structures. Systematic stimulation at 1–2 mA with each relevant tissue grasp provides continuous confirmation that neural elements are not incorporated between the instrument jaws before traction or application of energy, reducing the risk of occult traction or thermal injury (10, 23). This strategy is especially valuable in the posterior–lateral compartment of the thyroid lodge, where the RLN may enter the operative field in variable anatomical positions and branching patterns (Table 6).

**Table 6:** Operative technique: step-by-step SDI application. For each phase of thyroidectomy (lateral approach, RLN identification, active RLN dissection, Berry's ligament division, and final vagus assessment), the table specifies the SDI maneuvers, stimulation parameters, and corresponding EMG targets.

Step/phase	SDI use in surgical step	Stimulation/EMG target	Main SDI advantage
*Instrument connection and verification (V1)*	SDI connected to IONM via DIN 42802; initial vagus stimulation before thyroid manipulation.	Vagus 1–2 mA; EMG ≥100 µV confirms tube position, electrode contact, and circuit integrity.	Early functional integration of the dissecting instrument; rapid, tool-free V1 verification.
*Phase 1 – Lateral approach*	Blunt dissection and vessel isolation during middle vein division and lateral lobe mobilization.	Intermittent 1–2 mA with each relevant grasp to exclude nerve between jaws.	Continuous “safety scanning”; reduces occult traction/thermal injury in the posterior–lateral field.
*Phase 2 – RLN identification (R1)*	Cross-method mapping perpendicular/parallel to trachea to obtain R1.	Mapping at 1–2 mA; first reproducible RLN response defines course and variants.	Earlier functional RLN identification; narrows dissection zone; aids detection of non-recurrent RLN.
*Phase 3 – Active RLN dissection (R2)*	SDI used along RLN from thoracic inlet to cricothyroid joint with stimulation at each tissue contact.	1–2 mA; algorithm based on ≥100 µV stable, 50%–79% drop (pause), ≥80% drop/LOS (stop, V2).	Quasi-continuous monitoring; immediate traction-related EMG change detection and corrective action.
*Phase 4 – Berry's ligament*	SDI applied during Berry's ligament division, the highest-risk segment for RLN injury.	Repeated 1–2 mA; attention to focal amplitude reduction or weak conduction points.	Site-specific feedback in a critical zone; supports avoidance of focal conduction defects.
*Phase 5 – Final assessment (V2)*	Vagus restimulated with SDI or probe after hemostasis and before closure.	1–2 mA; V2 ≥ 100 µV (≈189–200 µV) without latency change predicts intact mobility.	High prognostic value; V2 loss triggers laryngoscopy and justifies staged surgery in bilateral cases.

### Phase 2: Inferior thyroid artery and RLN identification (R1)

Before direct visualization of the RLN, the SDI is applied perpendicular and then parallel to the tracheal axis (cross-method) to map the nerve's functional corridor and obtain the R1 signal. This electrophysiological navigation narrows the dissection field, facilitates targeted exposure, and aids intraoperative recognition of non-recurrent laryngeal nerve variants, which are reported in approximately 0.5%–1.5% of right-sided nerves at risk (10).

### Phase 3: active RLN dissection – core SDI application (R2)

This phase is the principal indication for SDI use. As the RLN is dissected free from surrounding fibro-adipose and fascial tissue along its course from the thoracic inlet to the cricothyroid joint, the SDI delivers stimulation with each tissue contact, while the IONM monitor continuously displays real-time EMG amplitude and latency. The surgeon can maintain uninterrupted visual focus on the operative field without repeated instrument exchange with a separate probe. In this context, the following decision algorithm is applied:

- EMG amplitude stable (≥100 µV with <50% variation from baseline): continue dissection.
- EMG amplitude decrease of 50%–79%: interrupt dissection, release thyroid traction, and observe for EMG recovery over 2–5 min; if recovery occurs, proceed cautiously. If the deficit persists, manage as loss of signal (LOS).
- EMG amplitude decrease ≥80% or frank LOS (<100 µV): immediately halt dissection, exclude technical failure with a V2 vagal check, localize the lesion site by sequential stimulation along the nerve, consider intravenous dexamethasone (4 mg) within 10 min, and, in bilateral procedures, consider staged completion surgery.

In the original prospective SDI series by Chiang et al., substantial EMG deterioration (>50% amplitude reduction) was observed in 19 of 168 RLNs, all attributable to traction stress during dissection (10, 26, 27). Prompt release of thyroid traction resulted in progressive EMG recovery in all affected nerves, and only one nerve with a 79% amplitude decrease developed transient postoperative vocal cord paresis; no permanent palsies were recorded. These findings underscore the ability of SDI-assisted monitoring to transform previously unmonitored intervals into actionable windows for timely corrective intervention (27).

### Phase 4: berry's ligament dissection

The ligament of Berry is the highest-risk segment of thyroidectomy, as the RLN courses in close proximity to or directly through the ligament in a substantial proportion of cases, often with complex branching that increases vulnerability to injury (28). Application of the SDI during Berry's ligament division provides continuous, site-specific electrophysiological feedback at this critical junction. In the Chiang et al. cohort, weak conduction points at the Berry's ligament region were identified in 10 of 168 RLNs on post-resection assessment, highlighting the pathophysiological relevance of this segment and the value of active monitoring during its dissection (10, 27).

### Phase 5: final assessment (V2) and wound closure

After achieving hemostasis and before wound closure, the SDI or a conventional hand probe is used to elicit the V2 response from the vagus nerve to assess final RLN function. A preserved V2 amplitude of at least 100 µV without significant latency prolongation provides a very high negative predictive value – reported up to approximately 99%–100% when using cutoffs around 189–200 µV – for intact postoperative vocal fold mobility (26). Conversely, loss of signal or a markedly reduced V2 amplitude requires immediate laryngoscopic evaluation and, in bilateral procedures, strongly supports considering a staged approach to avoid bilateral vocal fold paralysis.

## Special situations

### SDI in central and lateral neck lymphadenectomy

The clinical utility of SDI technology extends beyond thyroid lobectomy to compartment-oriented lymphadenectomy (29). In central neck dissection, the SDI enables simultaneous dissection and stimulation along the RLN throughout its course, providing high-frequency, maneuver-specific EMG feedback as fibro-lymphatic tissue is removed from the tracheoesophageal groove and paratracheal space. This continuous functional assessment facilitates early detection of traction or thermal stress during nodal clearance and helps maintain a safe dissection plane near the nerve.

In lateral neck dissection, the SDI provides a complementary advantage through continuous or high-density stimulation of the vagus nerve while nodal levels II–IV are dissected. Once the vagus is exposed and functionally verified, the SDI can deliver repeated low-intensity stimuli during stepwise clearance of the carotid–jugular axis, allowing real-time surveillance of global RLN function without interrupting the oncologic procedure. In this context, the ability to combine vagal monitoring and fine dissection in a single instrument reduces instrument exchanges, supports meticulous node removal around major vascular structures, and may lower the risk of unrecognized neurapraxia.

### SDI in reoperative thyroid and neck surgery

Reoperative procedures in the central or lateral compartment are characterized by dense fibrosis, scarring, and distorted anatomy, complicating identification of both the RLN and the vagus nerve (2). In such cases, placement of a circumferential vagal electrode for continuous monitoring may be technically challenging or undesirable due to the risk of additional manipulation of a nerve already at risk. SDI-assisted monitoring offers an alternative by enabling stepwise dissection and stimulation along scarred tissue planes without the need to encircle the vagus.

During reoperations, the SDI can probe fibrotic bands and adhesions with low-intensity stimulation before division, helping to distinguish neural tissue from avascular scar and reducing the likelihood of inadvertent transection or ligature. When the vagus nerve is difficult to expose safely, SDI-based mapping and serial RLN stimulation provide functional reassurance while avoiding excessive traction or skeletonization of the vagus. Consequently, SDIs are particularly valuable in reinterventions, where continuous, anatomy-guided neuromonitoring must be achieved under conditions of limited visibility and increased baseline neurophysiological risk.

### High-risk inflammatory thyroid disease

Thyroidectomy for Graves’ disease and Hashimoto thyroiditis is marked by significant vascularity, inflammatory edema, and dense capsular adhesions, all of which make it more difficult to maintain a true capsular dissection plane and increase the risk of RLN injury (2). In these cases, the SDI's ability to combine sharp and blunt dissection with systematic stimulation in a single instrument is particularly beneficial. Each adhesiolysis maneuver or capsular release can be preceded or accompanied by low-intensity stimulation, allowing the surgeon to test suspicious fibrotic bands and inflammatory tissue before division and thus distinguish scar tissue from neural structures in real time.

The continuous alternation of dissection and stimulation provided by the SDI is especially valuable around Berry's ligament and at the inferior thyroid pole, where inflammatory distortion often obscures the normal trajectory and branching pattern of the RLN. By delivering high-density, maneuver-specific EMG feedback without the need to repeatedly introduce a separate probe into an already crowded operative field, SDI-assisted monitoring helps stabilize the dissection plane on the true thyroid capsule and supports early identification and functional preservation of the RLN in both Graves’ disease and Hashimoto thyroiditis.

### Non-recurrent laryngeal nerve

The non-recurrent inferior laryngeal nerve (NRILN), present in approximately 0.5%–1.5% of right-sided nerves at risk and exceptionally on the left in association with major vascular anomalies, is one of the most hazardous anatomic variants in thyroid surgery (30). In this configuration, the nerve branches directly from the cervical vagus and courses horizontally toward the larynx without forming a recurrent loop, entering the operative field at an unpredictable, often more cranial level (30). These features markedly increase susceptibility to inadvertent transection, particularly when dissection follows standard expectations based on a recurrent course.

In NRILN cases, the use of conventional continuous IONM may be limited, because placement of a vagal electrode at the usual cervical level can fail to capture the true functional outflow if the NRILN branches proximally, above the site selected for the C-IONM clamp. In these circumstances, SDI-assisted mapping becomes particularly valuable: systematic stimulation with the SDI around the superior parathyroid gland, inferior thyroid artery, and upper pole region allows the surgeon to detect unexpected EMG responses and infer the presence of an NRILN before direct visualization. Once a suspicious early response is obtained, the dissection vector can be redirected cranially and medially, with meticulous SDI-guided stepwise exposure of the nerve from its origin on the vagus to its laryngeal entry point. In this way, SDI technology not only facilitates early intraoperative recognition of the NRILN but also offers a safer alternative to proximal vagal encirclement for C-IONM in anatomically unfavorable or uncertain situations (30).

## Role of SDI in minimally invasive approaches

### SDI in minimally invasive video-assisted thyroidectomy (MIVAT)

MIVAT, performed through a 15–20 mm cervical incision under endoscopic magnification, creates a narrow working corridor where instrument crowding and frequent exchanges are particularly disadvantageous (31). The SDI is inherently well suited to MIVAT and other mini-incision thyroidectomies because its slender, elongated shaft and compact tip are fully compatible with the limited access and with standard 5-mm trocar-equivalent ports, allowing it to function simultaneously as a dissector and stimulator without altering the standard setup. In contrast, conventional hand-held stimulators require repeated introduction and removal through the small incision or working channel, intermittently obstruct the endoscopic field, and further increase instrument traffic, which runs counter to minimally invasive principles. (Table 7)

**Table 7:** Role of SDI-based stimulation in minimally invasive and robotic thyroidectomy.

Approach/setting	Instrument type and connection	Monitoring concept	Key advantages over conventional hand-held probe/standard setup
MIVAT and mini-incision video-assisted thyroidectomy	Dedicated Beckdal SDI connected to IONM stimulator; slender, elongated shaft and compact tip compatible with 15–20 mm incision and 5-mm trocar-equivalent access.	Single instrument used for both dissection and stimulation within a narrow endoscopic corridor.	Reduces instrument exchanges and crowding; avoids repeated probe insertion that can obscure the endoscopic view; maintains minimally invasive workflow while adding integrated neuromonitoring.
TOETVA (transoral endoscopic thyroidectomy)	Standard long endoscopic forceps/dissectors adapted as “SDI-like” tools by connecting the stimulation cable where energy would normally be attached.	Maneuver-specific stimulation through 5-mm vestibular ports over a long working distance, with slightly increased current (≈0.3–0.5 mA above open thresholds).	Preserves usual endoscopic instrumentation and ergonomics; enables mapping and monitoring without dedicated SDI devices; minimizes additional hardware in an already constrained subplatysmal tunnel.
Robotic thyroidectomy (BABA, retroauricular, transoral)	Robotic forceps/dissectors rendered “SDI-like” by attaching the stimulation cable at the energy port; used in combination with percutaneous vagal electrode.	Hybrid paradigm: continuous vagal monitoring plus targeted, maneuver-specific stimulation along RLN/EBSLN via robotic instruments.	Allows functional surveillance while the surgeon is remote at the console; avoids extra robotic tools solely for stimulation; integrates neuromonitoring into standard robotic movements without workflow disruption.

Within the MIVAT environment, the SDI does not interfere with the endoscope or ancillary instruments; instead, it acts as a complementary device that integrates seamlessly into the existing operative choreography (31). Its matte finish and distinctive coloration ensure excellent visibility on video monitors without disruptive light reflection, facilitating precise dissection under magnification. Our Institutional experience indicates that SDI deployment in MIVAT is feasible without any modification of instrument design or port configuration and preserves the cosmetic and functional advantages traditionally associated with minimally invasive video-assisted thyroidectomy (31).

### Transoral endoscopic thyroidectomy (TOETVA)

TOETVA is among the most technically demanding scenarios for intraoperative neuromonitoring, as all maneuvers are performed through 5 mm vestibular ports over a long working distance from the oral entry site to the thyroid bed (11). In this context, commercially available Beckdal SDIs are not typically used; instead, standard endoscopic forceps and dissecting instruments routinely used for anatomic dissection are adapted to function as stimulating tools. Specifically, the cable that is ordinarily connected to an energy source (electrocautery or coagulation) is instead attached to the instrument shaft to deliver stimulation current, while the dissection technique remains unchanged (11).

Operative adaptations for TOETVA-IONM include the use of extended-shaft instruments (approximately 36–43 cm working length) to traverse the submental and subplatysmal tunnel, and a modest upward adjustment of stimulation intensity – generally by 0.3–0.5 mA above open-surgery thresholds – to compensate for the higher impedance associated with the longer, slimmer shaft and greater tissue interposition (11). Tip-end configurations are usually preferred over tip-lateral stimulation in the endoscopic setting, as they tend to achieve maximal EMG responses at lower currents. The neuromonitoring unit is positioned so that the visual display is directly visible to the assistant or scrub nurse, allowing continuous observation of EMG amplitude and latency while the primary surgeon maintains undivided focus on the endoscopic image (11).

### IONM with adapted stimulating instruments in robotic thyroidectomy

In robotic thyroidectomy – whether via bilateral axillo-breast approach (BABA), retroauricular, or transoral robotic access – the surgeon operates from a remote console, fundamentally altering the logistics of IONM (32). In these platforms, dedicated Beckdal SDIs are again not used; instead, the robotic forceps or dissecting instruments employed for standard anatomic manipulation can be rendered “SDI-like” by connecting the stimulation cable to the instrument at the point where an energy source would normally be attached. In this way, the same tool used for robotic dissection can deliver stimulation impulses without changing the robotic workflow (32).

Continuous IONM with a vagal electrode remains the preferred baseline strategy in robotic surgery, as it provides uninterrupted functional surveillance while the surgeon is remote. However, combining percutaneous C-IONM (such as an externally applied vagal clamp stimulator) with these SDI-type adapted robotic or endoscopic dissectors yields a hybrid paradigm with both continuous vagal monitoring and targeted, maneuver-specific stimulation along the RLN and EBSLN. Clinical series have reported high feasibility and safety of percutaneous C-IONM in large cohorts, with successful monitoring in all enrolled patients and no device-related complications, supporting the practicality of this combined approach for advanced robotic thyroidectomy platforms (32).

## SDI in parathyroid surgery

### RLN risk in parathyroid surgery

Although less common than in thyroid surgery, recurrent laryngeal nerve (RLN) injury remains a recognized complication of parathyroidectomy, with an incidence ranging from 0.5% to 3.9% in published series (33). The close anatomical relationship between the RLN and the parathyroid glands – posterior to the superior glands and anterior to the inferior glands – makes the nerve particularly susceptible during gland mobilization and hemostasis (33). In reoperative parathyroid surgery, adhesions and distorted anatomical landmarks further increase this risk. The RLN also serves as a critical anatomical reference during parathyroid exploration: superior parathyroid glands are typically dorsal to the nerve, while inferior glands are ventral. In this context, intraoperative electrophysiological mapping serves a dual purpose, providing both nerve protection and spatial orientation.

### Indications for IONM and SDI in parathyroid surgery

While the use of intraoperative neural monitoring (IONM) in parathyroid surgery is less standardized than in thyroidectomy, a growing body of evidence supports its selective application in high-risk situations, such as reoperations, intrathymic or retroesophageal glands, and minimally invasive approaches. The availability of a device capable of combining dissection and real-time stimulation around the target parathyroid gland is particularly advantageous in these scenarios. This integrated approach enhances both precision and safety, especially when managing superior parathyroid glands that are frequently in close proximity to or partially adherent to the RLN

### Minimally invasive parathyroidectomy and SDI

Minimally invasive video-assisted parathyroidectomy (MIVAP) is now standard technique for single-gland adenomas (34). As in MIVAP thyroidectomy, the restricted surgical corridor increases the value of SDI technology: it allows the surgeon to perform dissection while simultaneously confirming the position of the RLN, without instrument exchange or interruption of the operative flow (34). This is particularly relevant during mobilization of posteriorly located superior parathyroid adenomas that may be in close contact with the posterior thyroid capsule or the RLN, where SDI-guided dissection significantly improves procedural safety and nerve preservation

## EBSLN Monitoring and SDI

The external branch of the superior laryngeal nerve (EBSLN) innervates the cricothyroid muscle, and its injury leads to subtle but functionally significant impairment of high-pitch phonation and vocal projection (24, 35, 36). Intraoperative neuromonitoring (IONM) of the EBSLN, using cricothyroid muscle (CTM) twitch and/or electromyographic (EMG) responses, has been shown to be feasible in nearly all patients in prospective series using monopolar or bipolar stimulation (24, 35, 36). SDI technology can be configured to stimulate the EBSLN during superior thyroid pole management by delivering real-time stimulation to the superior thyroid artery and vein as they are dissected and mobilized (24, 35, 36). This allows progressive separation of the vascular pedicle from the nerve under continuous functional control, without the need for dedicated probe exchange. Additionally, SDI enables both initial (S1) and post-dissection (S2) testing, providing combined EMG and CTM twitch feedback, which is especially advantageous in the narrow, cranially located operative field of the superior pole (24, 35, 36). The slim, curved, atraumatic tip design allows precise work in this confined space while minimizing mechanical trauma to the nerve. Because EBSLN signal attenuation is associated with subtle but functionally significant voice changes — including loss of high-pitch capability, reduced dynamic range, and impaired projection that may be particularly disabling in professional voice users such as singers, teachers, and public speakers — its intraoperative preservation is clinically important. However, direct clinical evidence specifically quantifying the impact of SDI-assisted EBSLN monitoring on postoperative voice outcomes remains limited, as most available data derive from conventional monopolar or bipolar stimulation; SDI-assisted EBSLN monitoring should therefore be regarded as a promising extension of existing neuromonitoring frameworks rather than a fully validated practice, and future prospective, SDI-centered studies with detailed voice-quality endpoints are warranted.

## Evidence review

The published prospective evidence for SDI in open thyroid surgery derives primarily from Chiang et al. (2015; *n* = 100, 168 RLNs at risk). In this series, SDI use was feasible in 100% of cases with no device-related morbidity (10). Nineteen of 168 RLNs (11.3%) exhibited substantial EMG amplitude reduction (>50%) related to traction stress, with complete EMG recovery after traction release in all 19 nerves; 10 RLNs showed weak conduction zones at Berry's ligament after resection, and only one nerve with a 79% amplitude decrease developed transient postoperative vocal palsy, with no permanent palsies observed. These results compare favorably with historical outcomes from high-volume thyroid centers and underscore an important advantage over conventional intermittent monitoring, which cannot capture traction-induced EMG changes occurring between stimulations.

Additional clinical and experimental data further support the feasibility and effectiveness of stimulating dissecting concepts. Kim et al. evaluated an attachable ring stimulator (ARS) in 14 thyroidectomy patients (20 RLNs, 20 VNs) and demonstrated that ARS-evoked V1, R1, R2, and V2 EMG amplitudes were all >500 μV and statistically comparable to those obtained with a conventional stimulator, confirming equivalent stimulation efficacy and suitability for repeated intraoperative use (12). Zhang et al. tested an endoscopic stimulating dissector in a TOETVA porcine model (12 piglets, 24 RLNs, 24 VNs) and showed that the device allowed safe, real-time neuromonitoring with reproducible EMG responses and a clear dose–response relationship, at the cost of slightly higher stimulation thresholds than standard hand probes (11). Together, these studies indicate that stimulating dissecting instruments, whether open, attachable, or endoscopic, can reliably deliver neural stimulation comparable to conventional probes while integrating dissection and monitoring functions, thereby facilitating continuous feedback on RLN and VN integrity throughout thyroid surgery (Table 8) (10–12).

**Table 8:** Key evidence on stimulating dissecting instruments in thyroid surgery.

Study	Design/setting	Device	Key findings	Take-home message
Chiang et al., 2015 (Laryngoscope)	Prospective, open thyroidectomy	SDI (open)	100 pts, 168 RLNs; SDI feasible in all cases, no device morbidity; 19 RLNs with >50% EMG drop from traction, all reversible except 1 transient palsy; no permanent palsy.	SDI enables real-time detection and reversal of traction-related RLN stress during open surgery.
Kim et al., 2020 (Int J Endocrinol)	Clinical feasibility, open thyroidectomy	Attachable ring stimulator (ARS)	14 pts, 20 RLNs, 20 VNs; ARS EMG amplitudes (V1, R1, R2, V2) all >500 μV and statistically comparable to conventional probe.	ARS provides stimulation efficacy equivalent to a standard probe, with easier repeated stimulation.
Zhang et al., 2020 (Surg Endosc)	Experimental TOETVA pig model	Endoscopic SDI	12 piglets, 24 RLNs, 24 VNs; device feasible and safe; reliable EMG dose–response; slightly higher thresholds than hand probe.	Endoscopic SDI is feasible and safe for real-time RLN/VN monitoring in transoral endoscopic thyroidectomy.

## Learning curve and training

SDI-assisted IONM not only shortens the learning curve for neuromonitoring acquisition but also enhances the educational role of assistants and trainees (37–39). Trainees can perform dissection while simultaneously monitoring EMG responses, directly observing how meticulous and gentle maneuvers influence neural signals in real time. The supervising surgeon can independently observe EMG trends, intervene when adverse changes occur, and provide targeted, objective feedback based on quantifiable neurophysiological data rather than solely on subjective assessment. This dual dissection–monitoring paradigm has been successfully incorporated into resident training programs in high-volume endocrine surgery centers, where it supports progressive autonomy under robust functional safety monitoring.

## Practical implementation and future directions

### Instrument selection and procurement

From a practical perspective, stimulating dissecting instruments (SDIs) should be selected and implemented according to the technical infrastructure and workflow of each endocrine surgery unit. SDIs must be compatible with existing neuromonitoring platforms, ideally through universal DIN 42802 connectors that enable seamless integration with widely used monitoring systems. For configuration, curved-tip SDIs are generally suitable for standard thyroid dissection, while right-angle (Mixter-type) configurations are particularly advantageous for work around Berry's ligament and the posterior aspect of the thyroid lobe. Long-shaft variants are necessary for minimally invasive approaches such as MIVAP and TOETVA.

A key expectation among thyroid and parathyroid surgeons is the convergence of SDI technology with energy-based devices (EBDs), such as advanced bipolar and ultrasonic systems. The ideal future instrument would combine, in a single device, reliable neuromonitoring capability with the ability to dissect, coagulate, and divide tissue – eliminating the need to alternate between a separate stimulator, dissector, and energy device. Such “all-in-one” SDI-EBD platforms could further streamline operative workflow, reduce instrument exchanges, and enhance safety by providing continuous functional feedback precisely at the site where energy is applied.

From a health economic perspective, the potential cost-effectiveness of SDI adoption remains hypothetical and requires formal evaluation in dedicated cost–utility and cost–benefit studies. Although even modest reductions in postoperative vocal cord palsy rates might translate into substantial downstream savings through shorter hospital stays, reduced dysphonia management, and lower medicolegal risk, these assumptions have not yet been quantified in systematic economic analyses. At present, SDIs should therefore be considered an incremental-cost technology whose economic profile must be defined in future health economic research rather than presumed to be superior to conventional neuromonitoring.

Convergence of SDI technology with energy-based devices, robotic platforms, near-infrared fluorescence imaging, and artificial intelligence-assisted EMG interpretation represents an attractive conceptual trajectory. Prototype solutions combining dissection, coagulation, and stimulation, or adapting SDI-like tips to robotic instruments and NIRF-guided parathyroid identification, have been described at the preclinical and early clinical level, but robust multicenter data are currently lacking. These integrations should therefore be framed as emerging research directions rather than established clinical tools, and their true impact on safety, efficiency, and outcomes will need to be confirmed in prospective trials and large registries.

### Integration into operative protocols

For centers introducing SDIs, a stepwise implementation strategy is recommended. During the initial familiarization phase, SDIs can be used primarily for vagus (V1) and post-resection (V2) checks, while the conventional probe is retained for RLN stimulation (R1/R2), allowing the team to become accustomed to the ergonomics, cable management, and stimulation characteristics without altering the overall workflow. In the second phase, SDIs may be used for active dissection and R2 stimulation in straightforward primary thyroidectomies with favorable anatomy, progressively increasing the proportion of surgery performed under stimulation-guided conditions. Once both surgeons and staff have gained sufficient experience, full integration can be achieved, with SDIs serving as the primary dissecting and monitoring instrument throughout all critical phases of the procedure, and the standard probe reserved as backup for targeted stimulation in ambiguous or anatomically complex situations.

### Limitations and open questions

The current evidence base for SDI use still has several limitations. Most prospective clinical data come from a limited number of research groups, highlighting the need for independent multicenter series to confirm feasibility, safety, and effectiveness in diverse practice settings. No randomized controlled trial has yet directly compared SDI-assisted intermittent or continuous neuromonitoring with conventional probe-based neuromonitoring using RLN injury as a primary endpoint. Long-term outcomes, particularly permanent vocal cord palsy rates at 12 months, remain insufficiently characterized in SDI-based cohorts. Additionally, while prototype SDI solutions for robotic platforms have been described, dedicated, fully integrated robotic SDI systems are still at an early developmental stage, and robust clinical data in this area are lacking. Moreover, most prospective clinical series derive from a small number of high-volume endocrine surgery centers, underscoring the need for independent multicenter validation, and detailed voice-quality endpoints have not been systematically reported in SDI-based cohorts. Overall, SDI technology should therefore be regarded as a technically advanced and promising evolution of intraoperative neuromonitoring rather than a fully established standard of care, pending further high-level comparative and long-term evidence.

### Future directions

Future development of SDI technology is likely to intersect with several parallel innovations in endocrine surgery. These convergences — with energy-based devices, robotic platforms, near-infrared fluorescence imaging, and artificial intelligence–assisted EMG interpretation — currently remain conceptual, prototype-level directions supported by limited clinical data, and should be framed as emerging research priorities whose true impact on safety, efficiency, and outcomes will need to be confirmed in prospective, multicenter studies. One promising avenue is the integration of SDI with near-infrared fluorescence imaging, enabling simultaneous parathyroid identification and RLN monitoring in a single instrument pass. Another is the adaptation of SDI tips to robotic graspers and dissectors for use on platforms such as the da Vinci system, potentially combined with wireless transmission of EMG signals directly to the surgeon console. Additionally, real-time artificial intelligence–assisted analysis of EMG patterns could help differentiate true loss of signal from artifacts, offering automated decision support during SDI-guided dissection. Finally, development of cost-effective disposable SDI units may facilitate adoption in centers that have transitioned to predominantly single-use instrumentation pathways.

## Conclusions

The Stimulating Dissection Instrument (SDI) represents a technically advanced and clinically significant development in neuromonitoring for thyroid and parathyroid surgery. By integrating dissection and stimulation into a single device, it significantly reduces monitoring gaps caused by repeated exchanges between standard instruments and separate probes during critical nerve manipulation. Preclinical and clinical data show that SDI functions within safe and effective stimulation ranges and has not been linked to device-related neural morbidity. Traction-related EMG changes detected by SDI are consistently actionable and often reversible with prompt release. SDI is especially beneficial in minimally invasive and remote-access procedures (MIVAT, TOETVA, robotic surgery) and in complex parathyroid or reoperative cases, where limited exposure and anatomic distortion increase risk. As minimally invasive and robotic platforms become more common in thyroid and parathyroid surgery, dissection-coupled, high-density neuromonitoring with SDI-type instruments is likely to become essential for nerve preservation. Future research should focus on multicenter trials, dedicated robotic adaptations, and integration with emerging technologies such as NIRF parathyroid imaging and AI-assisted EMG interpretation.

## Data Availability

The original contributions presented in the study are included in the article/Supplementary Material, further inquiries can be directed to the corresponding author.

## References

[1] Francis DO Randolph G Davies L. Nationwide variation in rates of thyroidectomy among US medicare beneficiaries. JAMA Otolaryngol Head Neck Surg. (2017) 143(11):1122–5. 10.1001/jamaoto.2017.1746 PMID: 29049468; PMCID: PMC5710444. 29049468 PMC5710444

[2] Thomusch O Machens A Sekulla C Ukkat J Lippert H Gastinger I. Multivariate analysis of risk factors for postoperative complications in benign goiter surgery: prospective multicenter study in Germany. World J Surg. (2000) 24(11):1335–41. 10.1007/s002680010221 PMID: 11038203. 11038203

[3] Henry BM Graves MJ Vikse J Sanna B Pękala PA Walocha JA. The current state of intermittent intraoperative neural monitoring for prevention of recurrent laryngeal nerve injury during thyroidectomy: a PRISMA-compliant systematic review of overlapping meta-analyses. Langenbecks Arch Surg. (2017) 402(4):663–73. 10.1007/s00423-017-1580-y PMID: 28378238; PMCID: PMC5437188. 28378238 PMC5437188

[4] Chung TK Rosenthal EL Porterfield JR Carroll WR Richman J Hawn MT. Examining national outcomes after thyroidectomy with nerve monitoring. J Am Coll Surg. (2014) 219(4):765–70. 10.1016/j.jamcollsurg.2014.04.013 PMID: 25158909; PMCID: PMC4370365. 25158909 PMC4370365

[5] Dionigi G Barczynski M Chiang FY Dralle H Duran-Poveda M Iacobone M. Why monitor the recurrent laryngeal nerve in thyroid surgery? J Endocrinol Invest. (2010) 33(11):819–22. 10.1007/BF03350349 PMID: 21293170. 21293170

[6] Dionigi G Donatini G Boni L Rausei S Rovera F Tanda ML. Continuous monitoring of the recurrent laryngeal nerve in thyroid surgery: a critical appraisal. Int J Surg. (2013) 11(Suppl 1):S44–6. 10.1016/S1743-9191(13)60014-X PMID: 24380551. 24380551

[7] Dionigi G Van Slycke S Boni L Rausei S Mangano A. Limits of neuromonitoring in thyroid surgery. Ann Surg. (2013) 258(1):e1–2. 10.1097/SLA.0b013e318294559d PMID: 23673769. 23673769

[8] Schneider R Machens A Sekulla C Lorenz K Elwerr M Dralle H. Superiority of continuous over intermittent intraoperative nerve monitoring in preventing vocal cord palsy. Br J Surg. (2021) 108(5):566–73. 10.1002/bjs.11901 PMID: 34043775. 34043775

[9] Barczyński M Dworak M Krakowska K Pac A Konturek A. Clinical validation of NerveTrend versus NerveAssure mode of intraoperative neuromonitoring in prevention of recurrent laryngeal nerve injury during thyroid surgery: a randomized controlled trial. Ann Surg. (2025) 282(5):709–16. 10.1097/SLA.0000000000006872 PMID: 40758439; PMCID: PMC12513030. 40758439 PMC12513030

[10] Chiang FY Lu IC Chang PY Sun H Wang P Lu XB. Stimulating dissecting instruments during neuromonitoring of RLN in thyroid surgery. Laryngoscope. (2015) 125(12):2832–7. 10.1002/lary.25251 PMID: 25809677. 25809677

[11] Zhang D Li S Dionigi G Zhang J Wang T Zhao Y. Stimulating and dissecting instrument for transoral endoscopic thyroidectomy: proof of concept investigation. Surg Endosc. (2020) 34(2):996–1005. 10.1007/s00464-019-06936-2 PMID: 31218426. 31218426

[12] Kim J Moon HJ Chai YJ Lee JM Hwang KT Wu CW. Feasibility of attachable ring stimulator for intraoperative neuromonitoring during thyroid surgery. Int J Endocrinol. (2020) 2020:5280939–6. 10.1155/2020/5280939 PMID: 32411225; PMCID: PMC7204267. 32411225 PMC7204267

[13] Wang T Dionigi G Zhao Y Zhang D Pino A Dralle H. Tensile strength analysis of automatic periodic stimulation for continuous intraoperative neural monitoring in a piglet model. Sci Rep. (2021) 11(1):5898. 10.1038/s41598-021-84988-y PMID: 33723308; PMCID: PMC7960733. 33723308 PMC7960733

[14] Schneider R Randolph G Dionigi G Barczynski M Chiang FY Wu CW. Prediction of postoperative vocal fold function after intraoperative recovery of loss of signal. Laryngoscope. (2019) 129(2):525–31. 10.1002/lary.27327 PMID: 30247760. 30247760

[15] Schneider R Randolph G Dionigi G Barczyński M Chiang FY Triponez F. Prospective study of vocal fold function after loss of the neuromonitoring signal in thyroid surgery: the international neural monitoring study group’s POLT study. Laryngoscope. (2016) 126(5):1260–6. 10.1002/lary.25807 PMID: 26667156. 26667156

[16] Schneider R Machens A Randolph G Kamani D Lorenz K Dralle H. Impact of continuous intraoperative vagus stimulation on intraoperative decision making in favor of or against bilateral surgery in benign goiter. Best Pract Res Clin Endocrinol Metab. (2019) 33(4):101285. 10.1016/j.beem.2019.06.001 PMID: 31221571. 31221571

[17] Kauff DW Kronfeld K Gorbulev S Wachtlin D Lang H Kneist W. Continuous intraoperative monitoring of pelvic autonomic nerves during TME to prevent urogenital and anorectal dysfunction in rectal cancer patients (NEUROS): a randomized controlled trial. BMC Cancer. (2016) 16:323. 10.1186/s12885-016-2348-4 PMID: 27209237; PMCID: PMC4875600. 27209237 PMC4875600

[18] Kneist W Kauff DW Juhre V Hoffmann KP Lang H. Is intraoperative neuromonitoring associated with better functional outcome in patients undergoing open TME? Results of a case-control study. Eur J Surg Oncol. (2013) 39(9):994–9. 10.1016/j.ejso.2013.06.004 PMID: 23810330. 23810330

[19] Kneist W Kauff DW Lang H. Laparoscopic neuromapping in pelvic surgery: scopes of application. Surg Innov. (2014) 21(2):213–20. 10.1177/1553350613496907 PMID: 23892318. 23892318

[20] Skinner SA. Pelvic autonomic neuromonitoring: present reality, future prospects. J Clin Neurophysiol. (2014) 31(4):302–12. 10.1097/WNP.0000000000000055 PMID: 25083841. 25083841

[21] Carrano FM Iezzi L Melis M Quaresima S Gaspari AL Di Lorenzo N. A surgical instrument cover for the prevention of thermal injuries during laparoscopic operations. J Laparoendosc Adv Surg Tech A. (2019) 29. 10.1089/lap.2018.0742 PMID: 30698493. 30698493

[22] Andrychowski J Czernicki Z. Zastosowanie materiału izolacyjnego teflon–pladgets firmy genzyme–USA w operacjach konfliktu naczyniowo-nerwowego nerwu trójdzielnego application of the insulating material teflon–pladgets (genzyme-USA) during the microvascular decompression. Neurol Neurochir Pol. (2001) 35(Suppl 5):26–9. Polish. PMID: 11935676. 11935676

[23] Randolph GW Dralle H Abdullah H Barczynski M Bellantone R Brauckhoff M. Electrophysiologic recurrent laryngeal nerve monitoring during thyroid and parathyroid surgery: international standards guideline statement. Laryngoscope. (2011) 121(Suppl 1):S1–16. 10.1002/lary.21119 PMID: 21181860. 21181860

[24] Barczyński M Randolph GW Cernea CR Dralle H Dionigi G Alesina PF. International neural monitoring study group. External branch of the superior laryngeal nerve monitoring during thyroid and parathyroid surgery: international neural monitoring study group standards guideline statement. Laryngoscope. (2013) 123(Suppl 4):S1–14. 10.1002/lary.24301 PMID: 23832799. 23832799

[25] Bacuzzi A Dionigi G Del Bosco A Cantone G Sansone T Di Losa E. Anaesthesia for thyroid surgery: perioperative management. Int J Surg. (2008) 6(Suppl 1):S82–5. 10.1016/j.ijsu.2008.12.013 PMID: 19195946. 19195946

[26] Chiang FY Lee KW Chen HC Chen HY Lu IC Kuo WR. Standardization of intraoperative neuromonitoring of recurrent laryngeal nerve in thyroid operation. World J Surg. (2010) 34(2):223–9. 10.1007/s00268-009-0316-8 PMID: 20020124. 20020124

[27] Chiang FY Lu IC Kuo WR Lee KW Chang NC Wu CW. The mechanism of recurrent laryngeal nerve injury during thyroid surgery–the application of intraoperative neuromonitoring. Surgery. (2008) 143(6):743–9. 10.1016/j.surg.2008.02.006 PMID: 18549890. 18549890

[28] Liddy W Wu C-W Dionigi G Donatini G Giles Senyurek Y Kamani D. Varied recurrent laryngeal nerve course is associated with increased risk of nerve dysfunction during thyroidectomy: results of the surgical anatomy of the recurrent laryngeal nerve in thyroid surgery study, an international multicenter prospective anatomic and electrophysiologic study of 1000 monitored nerves at risk from the international neural monitoring study group. Thyroid. (2021) 31(11):1730–40. 10.1089/thy.2021.0155 PMID: 34541890. 34541890

[29] Liu X Zhang D Zhang G Zhao L Zhou L Fu Y. Laryngeal nerve morbidity in 1.273 central node dissections for thyroid cancer. Surg Oncol. (2018) 27(2):A21–5. 10.1016/j.suronc.2018.01.003 PMID: 29525322. 29525322

[30] Wang T Dionigi G Zhang D Bian X Zhou L Fu Y. Diagnosis, anatomy, and electromyography profiles of 73 nonrecurrent laryngeal nerves. Head Neck. (2018) 40(12):2657–63. 10.1002/hed.25391 PMID: 30466175. 30466175

[31] Dionigi G Boni L Rovera F Bacuzzi A Dionigi R. Neuromonitoring and video-assisted thyroidectomy: a prospective, randomized case-control evaluation. Surg Endosc. (2009) 23(5):996–1003. 10.1007/s00464-008-0098-3 PMID: 18806939. 18806939

[32] Lee HY Lee JY Dionigi G Bae JW Kim HY. The efficacy of intraoperative neuromonitoring during robotic thyroidectomy: a prospective, randomized case-control evaluation. J Laparoendosc Adv Surg Tech A. (2015) 25(11):908–14. 10.1089/lap.2014.0544 PMID: 26575249. 26575249

[33] Joliat GR Guarnero V Demartines N Schweizer V Matter M. Recurrent laryngeal nerve injury after thyroid and parathyroid surgery: incidence and postoperative evolution assessment. Medicine (Baltimore). (2017) 96(17):e6674. 10.1097/MD.0000000000006674 PMID: 28445266; PMCID: PMC5413231. 28445266 PMC5413231

[34] Miccoli P Berti P Materazzi G Massi M Picone A Minuto MN. Results of video-assisted parathyroidectomy: single institution’s six-year experience. World J Surg. (2004) 28(12):1216–8. 10.1007/s00268-004-7638-3 PMID: 15517483. 15517483

[35] Dionigi G Kim HY Randolph GW Wu CW Sun H Liu X. Prospective validation study of cernea classification for predicting EMG alterations of the external branch of the superior laryngeal nerve. Surg Today. (2016) 46(7):785–91. 10.1007/s00595-015-1245-9 PMID: 26362419. 26362419

[36] Iwata AJ Liddy W Barczyński M Wu CW Huang TY Van Slycke S. Superior laryngeal nerve signal attenuation influences voice outcomes in thyroid surgery. Laryngoscope. (2021) 131(6):1436–42. 10.1002/lary.29413 PMID: 33521945. 33521945

[37] Zhang D Brucchi F Barbieri D Kim HY Colombo C Donatini G. Simulation unlocked: transforming thyroid surgery training through high-fidelity and curriculum-based innovation-a systematic review. Langenbecks Arch Surg. (2026) 411(1):116. 10.1007/s00423-026-04013-6 PMID: 41814046. 41814046 PMC13056755

[38] Wu CW Randolph GW Barczyński M Schneider R Chiang FY Huang TY. Training courses in laryngeal nerve monitoring in thyroid and parathyroid surgery- the INMSG consensus statement. Front Endocrinol (Lausanne). (2021) 12:705346. 10.3389/fendo.2021.705346 PMID: 34220726; PMCID: PMC8253252. 34220726 PMC8253252

[39] Sun H Kim HY Carcoforo P Dionigi G. Cost and training are diffusion patterns limits for neural monitoring in thyroid surgery. Gland Surg. (2019) 8(4):334–5. 10.21037/gs.2018.11.08 PMID: 31538633; PMCID: PMC6723017. 31538633 PMC6723017

